# Counterproductive Work Behavior in Russian Nanotechnology Organizations

**DOI:** 10.11621/pir.2021.0105

**Published:** 2021-03-31

**Authors:** Karina Sayapina, Daniela N. Botone

**Affiliations:** a Financial University under the Government of the Russian Federation, Moscow, Russia; b Lucian Blaga University of Sibiu, Sibiu, Romania

**Keywords:** organizational behavior, counterproductive work behavior, organizational behavior management, nanotechnology

## Abstract

**Background:**

Organizational behavior plays a significant role in the effectiveness of enterprises specializing in nanotechnology. Its negative side – counterproductive work behavior (CWB) – has not been analyzed sufficiently in this industry. We evaluated different theoretical approaches to this problem.

**Objective:**

To estimate the predominant forms of counterproductive work behavior in relation to dimensions such as the intensity of the nanotechnology industry, seniority in the organization, and the age and gender of the subjects.

**Design:**

We used a descriptive exploratory methodology that analyzes the preponderance of counterproductive work behavior in profile companies throughout the Russian Federation. CWB was assessed through a self-report questionnaire and in-depth interview with each employee. The results were analyzed by correlation-regression analysis in SPSS.

**Results:**

We found significant correlations between the variables “intensity of the nanotechnology industry within the organization”, “seniority of employees within the organization”, “age of employees”, and the total score of CWB. Regarding the CWB dimensions, the highest average of the scores was obtained for “low level of conscientiousness” (mean = 21.75; SD = 2.9), followed closely by “low level of personal development” (mean = 20.53; SD = 3.09). Among the CWB dimensions, it seems that the conscientiousness of the employees plays a key role in the continuation of their professional activity and consequently in the increase of seniority in the organization.

**Conclusions:**

A professional difficulty can be perceived as a challenge by an employee with good physical and/or psychological resilience. Russian nanotechnology companies should evaluate their approach to dealing with employees and mitigate situations that might be unnecessarily stressful. From the data obtained through the semi-structured interview, we found that what happens in a work group is essential in the emergence of CWB. Organizations need clear policies that empower employees to deal with certain work tasks and with employees who engage in specific CWB.

## Introduction

Improvement of professional performance and responsible development of nanotechnology are goals of many organizations worldwide. Facilitating cross-disciplinary research has attracted much attention in recent years, with special concerns about nanotechnology working behavior (Bonaccorsi & Thoma, 2007; Gulumian, Verbeek, Andraos, Sanabria & Jager, 2016; Gravina et al., 2018; Khan, 2015; Kohnen, 2018; Ma et al., 2018; Maynard & Kuempel, 2005; Mogoutov & Kahane, 2007; Palmberg, Dernis, & Miguet, 2009; Schulte et al., 2014; Youtie, Iacopetta, & Graham, 2007; Zucker & Darby, 2007).

We notice that traditionally high qualifications and narrow specialization are at the forefront in nanotechnology companies in Russia: Most personnel (preliminarily those who work in enterprises in Moscow) know about the skills and experience required for the production process. Nanotechnology organizations in Moscow and the European part of Russia are interested in highly qualified engineers and managers who specialize in the solar energy sector, microprocessors production, and industrial-scale metal production.

Managing occupational safety and communicating risks to workers are the cornerstones of responsible nanotechnology. Since it is early in the commercialization of nanotechnology, there are still many unknowns and concerns; therefore, it is prudent to treat these issues as potentially hazardous until sufficient data are gathered for risk assessments.

## Counterproductive Work Behavior (CWB)

The problem of counterproductive work behavior has been insufficiently analyzed in the Russian Federation. Krushkova and Deviatovskaia (2017) highlighted the role of organizational vandalism in Russian organizations, including such forms as “vulgar” (primitive damage), “resource” (damage to the organizational resources), “information” (corporate sabotage), and “professional” (antisocial performance of professional activities).

In our study, we explored the problem of CWB in the nanotechnology industry, highlighting the most common counterproductive work behaviors in relation to employees’ seniority, the intensity of the nanotechnology profile, and some demographic characteristics of employees.

We have found no studies that describe a model of analysis for CWB in the Russian nanotechnology industry; therefore, we focused our attention on the most common types of counterproductive work behaviors found in other countries and organizations.

Counterproductive work behaviors are behaviors that are intended to harm the organization and are quite common among employees in many organizations worldwide; in most cases, these appear unnoticed or unreported (Bennet & Robinson, 2000). For this reason, the concept has played an essential role in Total Quality Management (TQM). Initially research was devoted to the question, “How do we achieve the best quality in the production process?” (Agrawal, 2019; Modgil & Sharma, 2016). Today, the TQM concept has been dramatically deepened, including such terms as organizational citizenship behavior and counterproductive work behavior, and it is connected with specific industries: Special rules are suggested, taking into account the specifics of an industrial sector, such as biotechnology or nanotechnology (Lavrynenko, Shmatko, & Meissner, 2018; Neyestani, 2016; Priede, 2012).

CWB includes acts directed against organizations and people; the most common CWB typology distinguishes between CWB targeted at the organization and CWB targeted at individuals (Bennett & Robinson, 2000).

The concept was defined as “voluntary behavior that violates significant organizational norms and in so doing, threatens the well-being of the organizations, its members, or both” (Robinson & Bennett, 1995, p. 556). Sackett and DeVore (2001) developed a hierarchical model of CWB: interpersonal deviance (harassment, gossip, verbal abuse, fighting) and organizational deviance (property deviance and production deviance). Property deviance consists of theft, property damage, and sabotage. Production deviance consists of absence, tardiness, long breaks, substance abuse, and sloppy work.

Gruys and Sackett (2003) found 11 categories of CWB: theft and related behavior, destruction of property, misuse of information, misuse of time and resources, unsafe behavior, poor attendance, poor quality of work, alcohol use, drug use, inappropriate verbal and physical actions. A few years later, Landy and Conte (2010) considered three common counterproductive behaviors: dishonesty, absenteeism, and sabotage. Dishonesty was defined as “employee theft of goods and theft of time (arriving late, leaving early, taking unnecessary sick days) or dishonest communications with customers, co-workers, or management” (Landy & Conte, 2010, p. 187). Absenteeism was defined as “a type of counterproductive behavior that involves failure of an employee to report for or remain at work as scheduled” (Landy & Conte, 2010, p. 188). Sabotage is “the intention to damage, disrupt, or subvert the organization’s operations for personal purposes of the saboteur, by creating unfavorable publicity, damage to property, destruction of working relationships, or harming of employees or customers”.

Employee theft is a major issue in many organizations worldwide (Cropanzano & Schminke, 2001; Greenberg, 2010; Landy & Conte, 2010; Murphy, 1993); for instance, a report by the American Management Association recorded a few decades ago that companies record huge losses each year, somewhere between $5 billion and $50 billion (Greenberg, 1990).

Greenberg (2002) reported that employee theft is correlated with moral development: “Employees who had attained Kohlberg’s conventional level of moral development refrained from stealing money when they worked in an office that had an ethics program in place. However, those at the preconventional level of development and who worked at an office without an ethics program stole from their employers” (Greenberg, 2002, p. 985).

Some researchers (Blau, 1985; Cropanzano & Schminke, 2001; Neuman & Baron, 2005) have identified another issue: The theft may be the effect of emotional states such as feelings of inequity and perceived violations of justice.

What are the organizational factors that cause absenteeism? In many studies (*e.g.,* Nicholson, Brown, & Chadwick-Jones, 1977; Nicholson & Johns, 1985) absenteeism is correlated with job commitment and job dissatisfaction, and it is caused by an informal agreement between a worker and a supervisor. Shamian and her colleagues (2003) suggested that stress perceived by the employees caused a high level of absenteeism, especially among women.

From another perspective, it seems that smoking plays an important role in producing CWB among workers: “Current smokers had significantly greater absenteeism than did never smokers, with former smokers having intermediate values; among former smokers, absenteeism showed a significant decline with years following cessation” (Halpern, Shikiar, Rentz & Khan,2001, 233). However, the problem of employees who are smokers can be much more complicated for a large organization. It is also possible that in addition to lost time as a result of illness, smokers are less productive at the workplace.

Sabotage (“the Lordstown Syndrome”) is strongly determined by stress and frustration among employees. In the early 1970s, in the General Motors company in the United States, workers intentionally dropped nuts and bolts into engines. Nowadays, it seems that injustice is the most common cause of sabotage. Researchers have shown that “when the source of injustice was interactional, individuals were more likely to engage in retaliation, and when the source of injustice was distributive, individuals were more likely to engage in equity restoration” (Ambrose, Seabright & Schminke, 2002, p. 947).

Other researchers have pointed out that perceived organizational support and the organizational ethical climate influence interpersonal deviance, whereby organizational justice and perceived organizational support affect organizational deviance among the support staff (Alias & Rasdi, 2015).

## CWB, Resilience, and Emotional Behavior

Turning to the Russian Federation, when a researcher tries to describe Siberian behavior in specific situations, it would be a significant mistake not to refer to what life in Siberia is like. Life and consequently work in Siberia are not easy, whether we are talking about physical work, intellectual work, or emotional work. Besides industrial development and climate, socioeconomic shocks to Siberian organizations include the collapse of the Soviet Union, which led to increased uncertainty about the region’s future economic development. Analyzing professional behavior in Siberia, some authors talk about “cultural resilience” (Crane, 2010; Forbes, 2013). According to Crane (2010, p. 2), cultural resilience is “the ability to maintain livelihoods that satisfy both material and moral (normative) needs in the face of major stresses and shocks; environmental, political, economic, or otherwise”. Therefore, when researchers try to perform an analysis of professional behavior in this geographical area, resilience, in all its forms, plays an important role.

In the Western world, many researchers have tried to highlight the roles of emotional behavior and resilience in CWB at the workplace. From a psychological point of view, resilience is a complex phenomenon that describes a fundamental coping competency (Seligman, 2012; Shoss, Jiang, & Probst, 2018; Sinclair & Wallston, 2004), associated with a variety of positive psychological and physical outcomes: professional performance, job satisfaction, work happiness, organizational commitment, organizational citizenship behavior, motivation, health, well-being, psychological contract, leadership style, self-esteem, and others.

Sinclair and Wallston defined resilience as the tendency to “cope with stress in a highly adaptive manner” (Sinclair & Wallston, 2004, p. 94). Highly resilient employees should generally be able to cope with a variety of stressors such as muscle pain caused by prolonged exertion or the psychological fatigue caused by intensive professional activity; this is rather a form of physical resilience. When we refer to psychological resilience, resilient workers seek out the positive in situations, search for creative solutions to difficult challenges, and focus on recovering losses they encounter (Bonanno, 2004; Tugade & Fredrickson, 2007).

The problem of resilience is very complex; usually resilient employees seek to adapt to difficult situations and tend to be more creative in teamwork; they also tend to be more conscientious and ambitious. Loyalty to the organization (as part of organizational citizenship behavior, OCB) can also be a true indicator of their professional behavior.

The assumption is that resilient employees tend to express organizational citizenship behavior more frequently, and CWB less often. Thinking in this direction, we suggest that resilience may be able to buffer the negative impact of CWB on a number of variables such as seniority, personality, and leadership style. We can talk about a “positive adaptation” that involves a lot of physical and emotional effort. A recent study has argued that resilience partially mediates the relationship between leadership style and sabotage, withdrawal, and theft, which are sub-dimensions of counterproductive work behavior (Ocel, 2018).

In our view, resilience is a very important predictor for diminishing or eliminating CWB, but it is not the only factor in this equation; for instance, there are few studies that try to outline the significance of emotional effort made by employees at the workplace.

In several studies, emotional effort was operationalized within the concepts of emotional work, emotion regulation, coping, emotional intelligence, occupational stress, emotional exhaustion, and others (Krischer, Penney & Hunter, 2010; Penney & Spector, 2005; Raman, Sambasivan, & Kumar, 2016; Spector & Fox, 2002; Spector & Fox 2005). According to Penney and Spector (2005), CWB has an instrumental use: It may be performed as an attempt to cope with stressful situations at work and reduce the experience of negative emotions. Although we have identified many studies that link CWB, resilience, and emotional behavior, unfortunately no study has demonstrated the specific influence of emotional effort on CWB in the nanotechnology industry.

There are also many studies that link CWB to a pattern of positive or negative emotions. Spector and Fox concluded in 2002: “Negative emotion will tend to increase the likelihood of CWB and positive emotion will increase the likelihood of OCB. CWB is associated with anger and anxiety, locus of control, and delinquency. OCB is associated with empathy and perceived ability to help (Spector & Fox, 2002, p. 269). This conclusion seems to be a bit restrictive, because there are jobs where anger can be operationalized as a positive emotion; for example, controlled anger may be a positive emotion for military personnel in special operations. Sadness or melancholy can have a positive effect on the creative process, whether we are talking about painters or musical composers.

The hypothesis that an emotion can change the valence of actions from negative to positive was supported by Krischer and colleagues, who outlined a positive effect of CWB in relation to some professional performance, an effect achieved through the instrumental role of emotion. Workers may experience some benefit as a result of CWB: “Employee withdrawal (*e.g.,* taking longer breaks than allowed) may reflect attempts by employees to limit their exposure to stressful situations and prevent subsequent strain. Production deviance (*e.g.,* intentionally working slowly) may serve as a strategy to gain control over stressors and the accompanying negative emotional reactions” (Krischer, Penney, & Hunter, p. 155).

Personality factors also have their well-defined role in the emergence of CWB (Barrick & Mount, 1991; Mount, Barrick, & Stewart, 1998; Salgado, 1997; 2003). Salgado (2003, p. 121) wrote: “Conscientiousness did not predict absenteeism and accident rate”. He concluded his study with a very interesting explanation, that “a possible explanation for the results is that accidents are by definition out of the volitional control of individuals and conscientiousness is largely a volitional trait”.

Self-control and the need for control over one’s working behavior is nuanced by Allen and Greenberger (1980). They suggested that individuals might engage in destructive or vengeful acts, including CWB, to increase feelings of control over a stressful situation. Also, it is very possible that those who perceive that they have control over their own professional actions will be willing to show more physical/intellectual/emotional effort and less CWB (Fox & Spector, 1999; Fox & Spector, 2006; Fox, Spector, & Miles, 2001; Spector, 1998; Spector & Fox, 2003; Spector et al., 2006).

We can conclude that CWB is related to a multitude of psychological constructs, such as physical and psychological resilience, emotions, personality factors, locus of control, and motivation. First we will try to identify forms of CWB in relation to the seniority of employees from two geographical areas characterized by economic development.

## Methods

We used a descriptive-exploratory methodology to identify possible forms of CWB in relation to employees’ seniority, the intensity of the company’s nanotechnology profile, and some demographic characteristics. For a start, we considered seniority in the organization as a significant indicator of organizational commitment, the opposite of CWB.

CWB was assessed through a self-report questionnaire and in-depth interview with each employee. Alternative sources for the assessment of CWB included objective indicators retrieved from organizational records, such as the KPI (key performance indicator) system for certain groups of workers. The KPI system makes it possible to attain transparency and clarity, fairness and perspective among personnel, which in turn directly influence loyalty to the organization. Other such indicators include monitoring of conflict situations (including resistance to innovations and lack of mutual understanding between workers) by the HR manager by means of expert assessment; and an employee card ID system, which is recognized as an effective instrument to tackle misbehavior by smokers and absenteeism.

### Participants

The participant population for the study comprised companies from the European and Asian areas of ​​Russia. A systematic random sampling procedure was first used to select the company from which the individual respondents were chosen. A variety of occupations were represented in this sample, the main groups of which were managers (50), engineers and technical specialists (39), research assistants (22), workers (18), students (7), entrepreneurs (6), public servants (5), and teachers (3). Most employees worked at privately owned companies. The sampling unit is made up of employees between 21 and 55 years of age. The sampling base was made up from lists of employees at the beginning of the year. They come mostly from Moscow and St. Petersburg in the European part of Russia, and Tomsk, Chelyabinsk, Barnaul, Kyshtym, and Ekaterinburg in the Asian part of Russia. The sample group comprised 150 employees (102 males, 48 females).

To sum up respondents’ portfolios, the main characteristics are presented in *Table 1*.

**Table 1:** Portfolio of respondents’ Russian nanotechnology organizations

Number of respondents	Number of employees	Ownership	Seniority	Type of activity	Intensity of nanotechnology	Specialization in nanotechnology
8	> 301	State	More than 48 months	Production, science and education, central coordination unit	Nanotechnology is one among other fields; there is also a science and education center	Huge spectrum (solar energy, nanocomposite material, optics, electronics, metallurgy)
62	101–300	State, private	More than 24 months,	Production, science and education	High intensity in nanotechnology, among other fields	Nanooptics, solar energy, microelectronics, nanomaterials, biotechnology
80	< 100	Private	Less than 24 months	Production, central coordination unit	Most are in nanotechnology industry only (33%); nanotechnology is one among other fields	Nanomaterials, biotechnology, electronics, optics

We should note that, although an organization can simultaneously work in different fields (production, science, education, central coordination unit), respondents could only identify the field in which they were working at present. All respondents were from organizations where nanotechnology intensity is highly developed (none of the firms were simply planning to develop nanotechnology, and none had no relationship at all to nanotechnology).

## Instruments

In investigating the CWB, we used a self-report survey and the semi-structured in-depth interview technique. The measure developed by Landy and Conte (2010) was chosen to determine and scale the CWB among the respondents. Participants were asked to rate how often they engaged in various counterproductive workplace behaviors, on a 5-point Likert scale ranging from Never (1) to Every day/Always (5). The survey consists of three sections: the first is devoted to common characteristics that link micro-level firm features and specialization in industry; the second section covers questions about counterproductive behavior and its opposite: organizational conformity–dishonesty, loyalty to the organization–organizational sabotage, conscientiousness–carelessness/negligence, and personal development–low commitment/ absenteeism; the last section identifies personal characteristics of the respondents (gender, working position, place of residence, number of employees, age of organization, and seniority in the organization). The reliability and validity of the survey were tested on a group of 67 employees from the private sector, before we started the research procedure. The internal consistency coefficients (Cronbach’s alpha) for each dimension are presented in *Table 2*. The test–retest reliability coefficient obtained a value of r_aa_ = 0.84, and it was achieved at an interval of three months between the two tests. Content validity was obtained by consulting four experts from the academic and economic sectors; they checked that the definition of the construct to be measured is clear and the items used to measure it are representative for the construct. The discriminant validity (r = –85) was obtained using a survey for evaluating organizational citizenship behavior, developed by van Dyne and LePine (1998), which was translated, adapted, and standardized for the Russian population.

**Table 2:** Alpha coefficient for CWB dimensions

	CWB dimensions	Alpha coefficient
1	Organizational conformity–Dishonesty	0.71
2	Loyalty to the organization–Organizational sabotage	0.84
3	Conscientiousness–Carelessness/Negligence	0.73
4	Personal development–Low commitment /Absenteeism	0.71

The semi-structured interview was used to complete the information obtained through the self-report technique. Questions in the semi-structured in-depth interview also included aspects of individual differences among personnel at work (whether managers divide workers by types of personality and form “harmonized” groups in carrying out projects; whether there are any conflict situations during work); handling negative emotions and high stress at work; methods of assessing degree of loyalty to the organization on the part of key highly qualified personnel; methods of motivating personnel to more effective work; expectations on the part of workers about their career development and their level of satisfaction with their current position; methods (predominantly expert assessment techniques) of monitoring internal communication among personnel; and finally, questions to identify special features of the organizational culture. The semi-structured in-depth interview technique was also applied to managers responsible for human resources in the nanotechnology organizations, who were asked additional questions about cases of counterproductive behavior among staff and ways of solving problematic situations.

### Procedure

After calling to confirm that the company met the sampling criteria, we personally delivered a questionnaire to the firm. Companies whose questionnaires had not been returned by the end of this procedure were considered non-respondents. Respondents completed the questionnaire individually in their own home or their work unit in one sitting, under the supervision of an interviewer. After completing the questionnaires, all the materials were passed to the interviewers and then to the authors to be analyzed. No personal information was recorded in the materials.

Prior to conducting the survey, participants were informed that the purpose of this study was to learn about their working behavior and social problems in their organization. All participants were assured that they were free to refuse participation if they did not agree with the objective of the study. Their confidentiality was also assured. Data collection occurred between October 10, 2019 and October 30, 2019.

For testing the research assumptions, we used several statistical methods processed in the SPSS 23 program.

### Hypothesеs

1. The intensity of the nanotechnology industry within the organization will be negatively related to measures of CWB.
2. The seniority of employees within the organization will be negatively related to measures of CWB.
3. The age of employees will be positively related to measures of CWB.

## Results

We conducted several statistical analyses to test our hypotheses. First, we aimed to establish that the intensity of the nanotechnology industry within the organization would be negatively related to measures of CWB (Hypothesis 1). We also assumed that the seniority of the employees within the organization would be negatively related to measures of CWB.

The total score of the CWB registered a mean of 49.22, with a standard deviation of 4.67. Regarding seniority in the organization (expressed in months), we obtained an average of 17.08, with a standard deviation of 8.46. The average age of the employees was 32.4 years, with a standard deviation of 3.75. The results showed significant correlations between the variables “intensity of the nanotechnology industry within the organization”, “seniority of employees within the organization”, “age of employees”, and the total score of CWB. We obtained a negative correlation between the variables CWB (total score) and “intensity of the nanotechnology industry within the organization” (r = -.252; p < .01); between the variables: CWB (total score) and “seniority of employees within the organization” (r = - .229; p < .01). These results confirm hypotheses no. 1 and no. 2. Also, the data show a significant correlation between variables CWB (total score) and the variable “age of employees” (r = .227; p < .01). This result does not confirm hypothesis no. 3 (that the age of employees would be positively related to measures of CWB).

According to our data, it seems that for the population of employees considered, we cannot say that their age has special significance in relation to counterproductive work behavior.

The results showed many interesting aspects regarding the dimensions of CWB and other variables. Although the number of employees investigated in the European and Asian parts of Russia was not equal (40 in Asia and 110 in Europe), the frequency of counterproductive work behavior among the employees was much higher in companies located in the European part of Russia.

Referring to dimensions of counterproductive work behavior (organizational conformity–dishonesty; loyalty to the organization–organizational sabotage; conscientiousness–carelessness, negligence; and personal development–low commitment/absenteeism), the results are as follows: employees from Russian companies located in Tomsk, Chelyabinsk, Barnaul, Kyshtym, and Ekaterinburg obtained a much lower score for organizational sabotage and dishonesty compared to their colleagues from European Russia (Moscow and St. Petersburg). Loyalty to the employer tends to be much higher, and consequently there was lower organizational sabotage, in the central Russian and Siberian companies. This score could be due to the characteristics of the population from Siberia. One key characteristic of the Siberian people is their high level of collectivism and mutual assistance: Cooperation, involvement, and success play important roles in relationships among personnel. The Siberian population is also characterized by very good physical and psychological resilience. Life where temperatures are frequently less than -30°C in the winter is not easy. Cooperation and involvement are very evident, both inside and outside a company. For example, no one is left stranded if a car has broken down on the highway. At very low temperatures, other drivers have a moral obligation to rescue passengers from the damaged car.

Tolerance toward violation of the norms and rules is low: Here, employees prefer to work in a coherent manner. Many employees from the Siberian companies surveyed in this study maintain principles of a so-called adjustment strategy: changing one’s own position and behavior in order to smooth out potential contradictions, frequently at the expense of one’s own expectations and personal interests. Specialists are more oriented to a long-term relationship in an organization than to a short-term one. Socio-emotional relationships between colleagues in the same department and/or other departments, as well as extra-professional activities among colleagues, are very important concepts in the perception of all employees in the Siberian companies we studied.

## Discussion

In order to prevent CWB, many managers responsible for this aspect periodically monitor the level of personnel satisfaction through surveys about corporate culture, the company’s values, the psychological state of workers, and relationships between workers and heads of departments.

In an Asian nanotechnology company that specializes in light-emitting diode production, personnel are interested in keeping appropriate norms of organizational behavior due to a grading system: All working positions are ranged according to their level of significance, difficulty, and amenability.

In the nanotechnology companies in the Asian region of Russia, respondents mentioned that appreciation and friendliness from their supervisor, and that person’s willingness to accept feedback, are the main conditions to feel satisfied and to fulfill all professional tasks.

The Asian Russian region is similar to collectivist cultural models: Appreciation of the supervisor is realized in context of formal and hierarchical organizational structure. Loyalty among personnel is predominantly high. The salary and award systems are directly related to qualifications.

However, although behavior of the employees is mainly characterized by cooperation and loyalty, the relationship between supervisors and subordinates is characterized by a low level of power distance. Managers prefer to use instruments of informal control, such as informal talks with personnel, to understand how fully and effectively projects are being performed. Working tasks are mainly unstructured in form, without strict goals and distribution of responsibility among personnel.

Specialists who have key positions in an organization have the opportunity to study on the job. The main roles of the HR manager in the Asian nanotechnology organizations in Russia include labor relations administration and conflict resolution.

For the companies located in European Russia, counterproductive work behavior is slightly different than for those in the Asian area.

First of all, personal development is more advanced than in Asian Russia. In the European companies, it is much more emphasized and is focused especially on economic attributes and less on human relations at work (professional relationships with a supervisor and/or co-workers). The amount of money earned monthly seems to be the most important factor in professional satisfaction or dissatisfaction.

In a nanotechnology company in Moscow specializing in solar energy projects, a manager pointed out that personnel satisfaction is provided by economic factors, because economic losses due to staff turnover amount to 7–10% of annual salary among laborers, 20–25% for highly qualified specialists, and up to 100% for supervisors. An innovative system of evaluating working conditions was another important way to tackle the problems of CWB: In all business units, specialized organizations helped to evaluate all workplaces to discover potentially harmful factors and possible deviations. As a result, a range of preventive activities was worked out, and this dramatically improved internal organizational behavior in that company.

The problem of tobacco smoking among personnel in nanotechnology enterprises is one of the key problems in the European part of Russia. Companies are trying to tackle it quite seriously: Most of our respondents mentioned that the most popular instrument here is a strict punishment system (prohibition of smoking on company premises, “a fully healthy law”). One respondent pointed out that he could not handle such strict rules and wrote a letter of resignation; but considering his high level of qualification and narrow specialization (he was a nanotechnology material designer), his general manager allowed him to smoke one cigarette per day at lunchtime, but with a deduction of 5% from his salary. As a result, the respondent has been extremely motivated to quit smoking.

As we have mentioned, representatives of a huge state nanotechnology company from Moscow were among our respondents. This company found a quite extraordinary approach to solve the smoking problem: Apart from a complex system to support non-smoking workers (including both tangible and intangible motivations – bonuses, higher salaries, a board of honor, corporate events), it has invested more than $50 million in research and development of an anti-nicotine vaccine (in partnership with American scientific centers). This vaccine is aimed at the human immune system activation: Nicotine is directly connected with neuron receptors, that is why it gives immediate pleasure to a smoker. The whole production cycle of the vaccine is planned to be fully realized in Russia. In our opinion, this measure is one of the top ones to get personnel to quit smoking.

In European Russia, the main characteristic of the companies we surveyed is the so-called short-term career; according to the information we gathered from the semi-structured interview, staff turnover here is higher than in Asian Russia.

Personnel in the European companies do not show significant loyalty towards the organization, and the key instrument for motivation of an individual is financial (estimation of how talented a specialist is, what innovation he/she has offered to supervise, how many projects he/she has already participated in). Here, the nanotechnology organizations prefer to comply with formal control instruments. Hiring of personnel is predominantly based on work experience.

**Figure 1.**
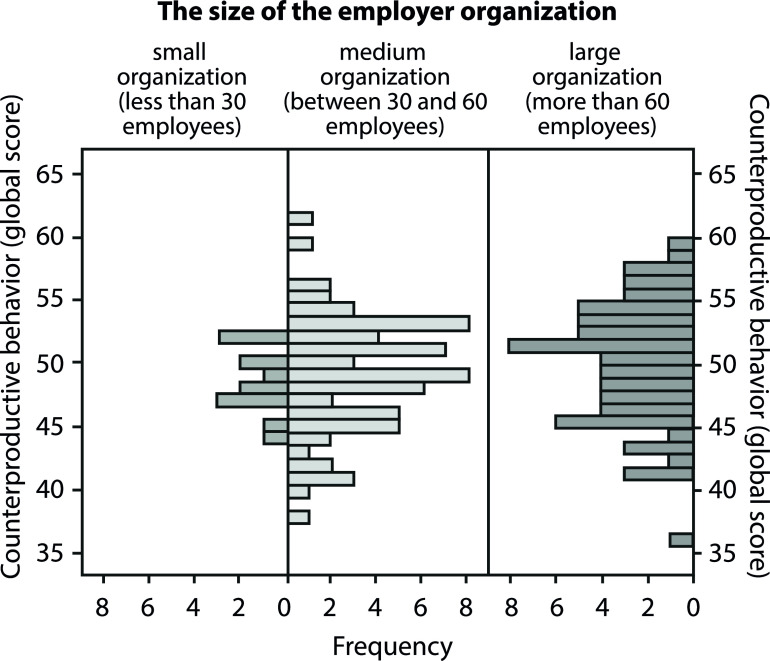
Frequency of CWB according to the size of the organization

However, we cannot say that the place where the employee lives or the geographical area where the company is located are the most important factors associated with a high frequency of counterproductive work behavior. As can be seen in *Figure 1*, the size of the organization seems to be a factor that associated with CWB.

Although the frequency of CWB is higher in organizations with more than 60 employees, our data do not allow us to say for sure that the size of the company is a causal factor in increasing CWB frequency among employees. The average employee‘s age was 32.4 years, and the average professional seniority was 17.08 months (*Table 3*).

**Table 3:** Descriptive statistics (mean and SD) for the research variables

	Mean	Standard Deviation	N
Age of employee (years)	32.44	3.751	150
Where the employee lives	1.05	.225	150
Size of the organization	2.38	.642	150
Seniority in organization (months)	17.08	8.466	150
Seniority in profession (months)	49.08	29.897	150
Counterproductive behavior (total score)	49.22	4.674	150
Intensity of nanotechnology	3.16	1.165	150
Gender	1.32	.468	150

Regarding the CWB dimensions, the highest average of the scores was obtained in the case of “low level of conscientiousness” (mean = 21.75; SD = 2.9), followed closely by “low level of personal development” (mean = 20.53; SD = 3.09) (*Table 4*).

**Table 4:** Descriptive statistics (mean and SD) for the CWB dimensions

	Mean	Standard Deviation	N
Seniority in organization (months)	17.08	8.466	150
Counterproductive work behavior (total score)	49.22	4.674	150
Counterproductive work behavior (low level of organizational conformity)	10.13	2.086	150
Counterproductive work behavior (low level of loyalty to the organization)	6.67	1.701	150
Counterproductive work behavior (low level of personal development)	20.53	3.091	150
Counterproductive work behavior (low level of conscientiousness)	21.77	2.904	150
Intensity of nanotechnology	3.16	1.165	150

**Figure 2.**
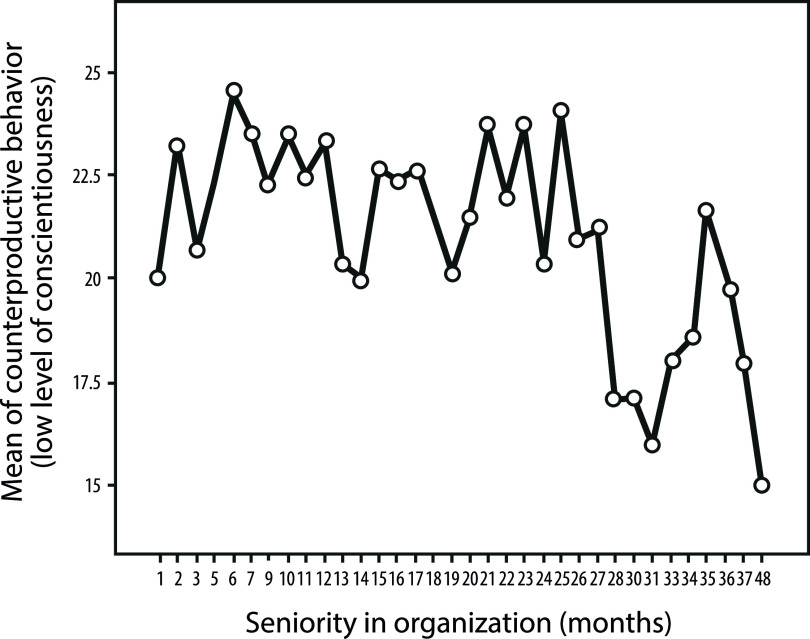
Low conscientiousness depending on seniority in the organization (number of months)

**Figure 3.**
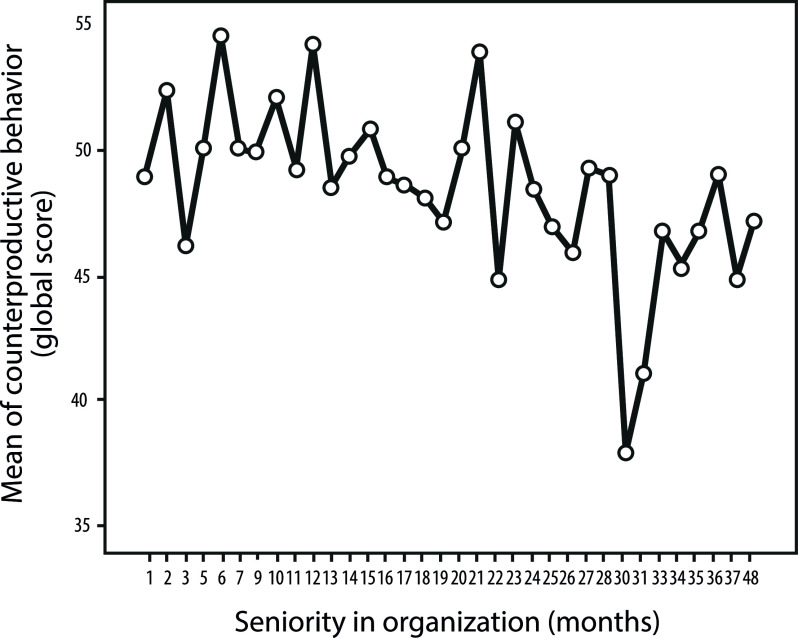
Frequency of CWB depending on seniority in the organization (number of months)

As can be seen in *Figure 2* and *Figure 3*, CWB tends to decline as the number of months in the organization increases. Among the CWB dimensions, it seems that the conscientiousness of employees plays a key role in their continuation of the professional activity and consequently the increase of their seniority in the organization. As can be seen in Figure 3, as the seniority increases (measured in number of months), so does conscientiousness at the workplace. The high score in Figure 2 represents the level of counterproductive work behavior, characterized mainly by a low level of conscientiousness (a high level of negligent and careless behavior at work).

## Conclusions

The emergence of counterproductive work behavior in Russian nanotechnology companies can be related to a multitude of factors: the size of the company, the leadership style, or the organizational culture. Employee age is not a significant factor.

Personality plays an important role as well, as the interplay of individual differences and the work environment combine to induce specific forms of CWB. It is well known that negative emotions are significant in much CWB, but the effects of negative emotion at the workplace may be directly conditioned by the employee’s physical and psychological resilience. Thus, a professional difficulty may be perceived as a challenge by an employee with good physical and/or psychological resilience, leading the employee to become more professionally involved, to show more loyalty to the organization, and as a result to obtain higher seniority.

Two types of employees have emerged from our study: the eastern Russian employee, generally with good physical and psychological resilience, for whom altruism and loyalty to the company are very important issues and also conditions for professional success; in the western part of Russia (especially in Moscow), we identified a typical employee who is very determined to achieve professional success, but who is experiencing frustration regarding money, position in the organization, or lack of control at work. Also, we repeatedly identified specific forms of CWB in this area. Basically, in the western part of Russia, we cannot talk about “positive adaptation” in most cases, adaptation that involves a great physical and emotional effort from the employee. For example, in the western area, often work stressors can trigger anger or anxiety that under some work circumstances leads to CWB.

Russian nanotechnology companies should evaluate their approach to dealing with employees and mitigate situations that might be unnecessarily stressful. From the data obtained through our semi-structured interview, what happens in a work group is essential in the emergence of CWB. Organizations need clear policies that empower employees to deal with certain work tasks and with employees who engage in specific behaviors and CWB.

## Limitations

The number of participants is a limitation of our study. We set out to have a larger number, but certain objective conditions prevented us from obtaining more participants.
